# Associations of statins and antiretroviral drugs with the onset of type 2 diabetes among HIV-1-infected patients

**DOI:** 10.1186/s12879-016-2099-5

**Published:** 2017-01-07

**Authors:** Vincenzo Spagnuolo, Laura Galli, Andrea Poli, Stefania Salpietro, Nicola Gianotti, Piermarco Piatti, Francesca Cossarini, Concetta Vinci, Elisabetta Carini, Adriano Lazzarin, Antonella Castagna

**Affiliations:** 1 Department of Infectious Diseases, San Raffaele Scientific Institute, via Stamira d’Ancona 20, 20127 Milan, Italy; 2 Università Vita-Salute San Raffaele, Milan, Italy; 3 Cardiometabolic and Clinical Trials Unit, Internal Medicine Department, Metabolic and Cardiovascular Division, San Raffaele Scientific Institute, Milan, Italy

**Keywords:** Diabetes, Statins, HIV, Antiretrovirals drugs, Risk factors, Cohort study

## Abstract

**Background:**

Statin use is associated with a modest increase in the incidence of type 2 diabetes mellitus (DM) among the general population. However, HIV-infected patients have a higher risk of developing DM, and it is unclear whether statins have a diabetogenic effect in these patients. Therefore, we investigated the associations of statin use and exposure to antiretroviral drugs with type 2 DM onset in a cohort of HIV-infected patients.

**Methods:**

This retrospective, controlled, cohort study identified HIV-1-infected patients who did not have DM and were not receiving statins at their antiretroviral treatment (ART) initiation. Follow-up was accrued from ART initiation to the earliest instance of a DM diagnosis, loss to follow-up, death, or last available visit. The incidence of DM was estimated according to statin use, which was adjusted for periods without statin treatment. The Fine-Gray competing risk model was used in the multivariate analysis to identify risk factors for developing DM.

**Results:**

The analyses evaluated 6,195 patients followed for 9.8 years (interquartile range: 4.3–16.3 years). During 64,149 person-years of follow-up (PYFU), 235 patients developed DM (crude incidence: 3.66 [95%CI: 3.20–4.13] per 1,000 PYFU), and 917 (14%) patients used statins. After adjusting for potential confounders, statin use was associated with a non-significant increase in the risk of DM (AHR: 1.21, 95% CI: 0.71–2.07; *P = 0.47*). DM was more likely among patients who were ever treated with stavudine, and less likely among those ever treated using emtricitabine, tenofovir, abacavir, efavirenz, nevirapine, atazanavir or darunavir.

**Conclusions:**

A higher risk of diabetes mellitus was not associated with statin treatment but with traditional risk factors and stavudine use while a reduced risk of DM was associated with the use of emtricitabine, tenofovir, abacavir, efavirenz, nevirapine, atazanavir or darunavir.

**Electronic supplementary material:**

The online version of this article (doi:10.1186/s12879-016-2099-5) contains supplementary material, which is available to authorized users.

## Background

Hypercholesterolemia is common among HIV-infected patients who are receiving antiretroviral therapy (ART) [1] and can contribute to the increased risk of cardiovascular disease in this population [2, 3]. Therefore, 3-hydroxy-3-methylglutaryl-coenzymeA (HMG-CoA) reductase inhibitors (statins) are recommended [4] and extensively prescribed to treat hypercholesterolemia and to prevent cardiovascular events among HIV-infected patients. In additional to their cholesterol-lowering properties, statins have recently been associated with reductions in overall mortality [5–7], the risk of non-AIDS malignancies [7, 8] and the risk of AIDS-defining malignancies [8, 9] among HIV-infected patients.

In contrast, concerns have recently been raised regarding glucose metabolism impairment among statin users, with different studies in the general population revealing a possible association between statin use and the risk of incident type 2 diabetes mellitus (DM) [10, 11], as well as a dose-related effect [12]. Although the mechanisms underlying this increased risk of incident DM are not fully understood, Mendelian randomization studies [13] suggest that they are related, at least in part, to reduced HMG-CoA reductase activity [14]. Therefore, the possible diabetogenic effect of statins may be particularly relevant among HIV-positive patients, who have a higher risk of developing DM, compared to the general population [15].

Unfortunately, there are few studies regarding statin use and incident DM among HIV-infected patients, and these studies have reported not univocal results. For example, a randomized study [16] of 72 patients reported that rosuvastatin use was associated with increased insulin resistance but not with a clinical diagnosis of DM and a recent italian study did not observe any association between statins use and DM risk [17]. In contrast, the HOPS cohort study [18] found a slightly increased risk of incident DM among patients who were treated using statins. Therefore, the present study aimed to evaluate the associations of statin use and exposure to antiretroviral drugs with the onset of type 2 DM in a large cohort of HIV-infected patients.

## Methods

This retrospective observational study was approved by the ethics committee of the San Raffaele Scientific Institute, and examined patient data from the Infectious Diseases Department database at the San Raffaele Hospital (IDD-HSR). This observational database collects demographic, clinical, therapeutic, and laboratory data from adult patients who are receiving primary inpatient or outpatient care for HIV infection at the Infectious Diseases Department of the San Raffaele Scientific Institute (Milan, Italy). At their first visit to the clinic, the patients provide written informed consent for scientific analysis of their clinical and laboratory data. Information regarding the prescribed antiretroviral and concomitant drugs (type, dosage, and dates of start and stop) are prospectively recorded at each visit by the treating physician, and these data are subsequently checked by skilled data managers. However, patient adherence to the prescribed drugs is not routinely assessed.

In the present study, we included adults who were infected with HIV-1 and subsequently started ART between January 1991 and November 2014. These patients were not using statins, had not been diagnosed with type 2 DM at the start of ART, and had undergone at least one test to determine their fasting glucose and lipid levels after starting the ART (i.e., one follow-up examination in addition to that of baseline). Details of the patients’ selection process is illustrated in the Additional file 1: Figure S1.

We diagnosed DM according to the American Diabetes Association criteria [19], based on two consecutive fasting plasma glucose levels of ≥126 mg/dL, or a 2-h plasma glucose level ≥200 mg/dL during an oral glucose tolerance test, or two consecutive fasting glycated hemoglobin levels of ≥48 mmol/mol, or a prescription for any antidiabetic medication [the median (IQR) number of fasting glucose determinations per patient was 29 (13–50)]. An expert physician (VS) and a diabetologist (PP) reviewed all diagnoses to verify the data’s plausibility and completeness before the analyses. We excluded patients with a diagnosis of type 2 DM before the initiation of ART (prevalent cases).

In the present study, all statin treatments were started after the initiation of ART, and preceded the diagnosis of DM (if applicable). Patients were required to receive a statin for at least 30 days to be considered a statin user. To evaluate the effect of statin dose on the incidence of DM, we considered the following dose categories [11]: high dose (rosuvastatin at ≥40 mg or atorvastatin at ≥80 mg), moderate dose (rosuvastatin at 10–40 mg, atorvastatin at 20–80 mg, or simvastatin at ≥80 mg), and low dose (rosuvastatin at <10 mg, atorvastatin at <20 mg, simvastatin at <80 mg, or fluvastatin and pravastatin at any dose); patients were categorized according to the highest dose category recorded during follow-up.

Follow-up was counted from the date of starting ART (baseline) to the earliest instance of a type 2 DM diagnosis, death, loss to follow-up, or last available visit.

Results were reported as median (interquartile range [IQR]) or frequency (%), as appropriate. The baseline characteristics of HIV-infected patients according to statin use were compared using the chi-square test for categorical variables and the Wilcoxon rank-sum test for continuous variables. Crude rates of incident type 2 DM were reported as events per 1,000 person-years with the exact 95% Poisson confidence intervals (CIs) among the entire sample, non-statin users, and statin users (the calculation for statin users accounted for their periods of non-use) [20].

The effect of death as an event competing with the onset of type 2 DM was modeled using the Fine-Gray model [21], and factors that were associated with the onset of type 2 DM were identified using subhazard ratios (a measure of relative risk that considers death). No major violations of the proportional-hazards assumption were detected using the interactions terms of the predictors as a function of time. The multivariate model (Model 1) included characteristics with a *p*-value of ≤ .20 in the univariate analyses or factors that are commonly associated with DM (e.g., smoking or hepatitis C virus [HCV] co-infection) in addition to the calendar year of ART initiation. We classified patients as smokers if they had smoked at least once, and patients who were anti-HCV-positive based on at least one positive HCV-antibody test result. Patients were categorized according to the highest BMI recorded during the follow-up. Race was not included in the multivariate models, as almost all patients (with or without DM) were white. All ART drugs that were used by at least 25% of the patients and darunavir (used by 23%) were also included in the multivariate models (regardless of their *p*-value in the univariate analyses) to investigate the association between these drugs and DM occurrence. The use of ritonavir included both full and boosting doses. Statin use was considered as a time-updated variable: patients who had a history of statin prescriptions during the follow-up were alternatively considered over time as either non-statin users or statin users depending on whether they were receiving the treatment or not. The other time-updated covariates were age, CD4+, HIV RNA, hemoglobin levels, and fasting levels of glucose and triglycerides. A second multivariate model accounted for the effect of an additional risk factor (Model 2) including high-density lipoprotein (HDL)-cholesterol (time-updated variable), as this factor is independently associated with new-onset DM [22].

All statistical tests were two-sided at the 5% level and were performed using SAS software (version 9.2; SAS Institute, Cary, NC).

## Results

We evaluated data from 6,195 patients, who included 4,828 (78%) men. The median age at the end of follow-up was 47.2 years (IQR: 40.6–52.7 years), the median time since the first HIV-positive result was 15 years (IQR: 8–21 years), and the median duration of ART was 10 years (IQR: 4–16 years). Approximately 27% of the patients exhibited HCV-positive serology results, 6% of the patients had a reactive HBV surface antigen. The median nadir CD4+ cell count was 283 cells/μL (IQR: 162–408 cells/μL), 10% of the patients were diagnosed with AIDS before initiating ART, 28% of the patients were smokers, and the median BMI was 23.0 kg/m^2^ (IQR: 21.1–25.7 kg/m^2^). The median baseline laboratory results were total cholesterol levels of 157 mg/dL (IQR: 132–184 mg/dL), low-density lipoprotein (LDL) cholesterol levels of 100 mg/dL (IQR: 80–121 mg/dL), HDL-cholesterol levels of 38 mg/dL (IQR: 32–45 mg/dL), triglyceride levels of 104 mg/dL (IQR: 75–152 mg/dL), fasting plasma glucose levels of 85 mg/dL (IQR: 79–92 mg/dL), CD4+ cell counts of 307 cells/μL (IQR: 174–442 cells/μL), and log_10_ HIV RNA levels of 4.74 (IQR: 4.16–5.20). During the follow-up, 97% of the patients used nucleoside reverse transcriptase inhibitors (NRTIs), 79% of the patients used protease inhibitors (PIs), 61% of the patients used non-nucleoside reverse transcriptase inhibitors, 12% of the patients used integrase strand transfer inhibitors, and 9% of the patients used entry inhibitors.

During 64,149 person-years of follow-up, 235 patients developed DM, which corresponded to a crude incidence rate of 3.66 per 1,000 person-years of follow-up (95% CI: 3.20–4.13 per 1,000 person-years of follow-up). The patients’ characteristics according to DM status are reported in Table 1. Patients with new-onset DM were more likely to be older, male, overweight or obese, co-infected with HCV or HBV, exhibit prolonged HIV infection, and have a lower CD4+ cell count nadir. When we analyzed the exposures to ART drugs according to the occurrence of DM (Additional file 2: Table S1), we observed that patients with DM were more likely to be exposed to “first-generation” NRTIs (e.g., zidovudine, stavudine, didanosine and lamivudine) or PIs (indinavir, nelfinavir, and saquinavir), compared to patients without DM.

**Table 1:** Patients characteristics according to occurrence of type 2 diabetes

Characteristics	Type 2 diabetes (*n* = 235)	No type 2 diabetes (*n* = 5960)	*p*-value
Age (years), median (IQR)	40.3 (35.6–50.0)	35.3 (30.2–41.6)	<0.001^a^
Males, *n (%)*	201 (86%)	4627 (78%)	0.002^b^
White Race, *n (%)*	224 (95%)	5542 (93%)	0.16^b^
Smoke, *n (%)*			0.96^b^
Current/ex-smoker	99 (42%)	2384 (40%)	
Never	75 (32%)	2086 (35%)	
Unknown	61 (26%)	1490 (25%)	
Body Mass Index, *n (%)*			<0.001^b^
Normal (<25 kg/m2)	83 (35%)	2463 (41%)	
Overweight (25–29.9 kg/m2)	69 (29%)	1425 (24%)	
Obese (≥30 kg/m2)	34 (15%)	355 (6%)	
Unknown	49 (21%)	1717 (29%)	
HIV risk factor, *n (%)*			0.015^b^
IVDU	46 (20%)	1134 (19%)	
MSM	54 (23%)	1893 (32%)	
Heterosexual	49 (21%)	1225 (21%)	
Other/Unknown	86 (37%)	1708 (29%)	
Ab-anti HCV, *n (%)*			0.014^b^
Positive	83 (35%)	1617 (27%)	
Negative	135 (58%)	3729 (63%)	
Unknown	17 (7%)	613 (10%)	
HBsAg, *n (%)*			0.004^b^
Positive	21 (9%)	323 (5%)	
Negative	197 (84%)	4853 (82%)	
Unknown	17 (7%)	784 (13%)	
Years since first HIV-positive test, median (IQR)	19.0 (14.4–25.6)	14.4 (7.7–20.9)	<0.001^a^
AIDS diagnosis before ART, median (IQR)	29 (12%)	599 (10%)	0.27^b^
Nadir CD4 cell count before ART (cells/μL), median (IQR)	231 (91–395)	285 (165–408)	0.019^a^
Baseline Total Cholesterol (mg/dL), median (IQR)	153 (127–198) (available in 126 pts)	157 (132–183) (available in 2271 pts)	0.92^a^
Baseline HDL-cholesterol (mg/dL), median (IQR)	35 (31–40) (available in 6 pts)	38 (32–45) (available in 1056 pts)	0.53^a^
Baseline LDL-cholesterol (mg/dL), median (IQR)	98 (84–114) (available in 4 pts)	100 (80–121) (available in 985 pts)	0.87^a^
Baseline Fasting Glucose (mg/dL), median (IQR)	96 (85–106) (available in 51 pts)	85 (79–92) (available in 2626 pts)	<0.001^a^
Baseline Triglycerides (mg/dL), c	144 (116–206) (available in 43 pts)	103 (75–150) (available in 2324 pts)	<0.001^a^
Baseline CD4 cell count (cells/μL), median (IQR)	240 (99–422) (available in 126 pts)	307 (177–443) (available in 3557 pts)	0.018^a^
Baseline HIV-RNA (log_10_ copies/ml), median (IQR)	4.78 (4.17–5.31) (available in 76 pts)	4.74 (4.16–5.20) (available in 2929 pts)	0.65^a^
Baseline haemoglobin (mg/dl), median (IQR)	13.7 (11.9–15.1) (available in 61 pts)	14.0 (12.7–14.9) (available in 2883 pts)	0.63^a^
Baseline creatinine (mg/dl), median (IQR)	0.87 (0.72–0.96) (available in 34 pts)	0.82 (0.71–0.93) (available in 2205 pts)	0.70^a^

Baseline: date of ART initiation; if not indicated the characteristic is intended at the censoring date

^a^ by Wilcoxon rank sum test

^b^ by Chi-square or Fisher exact test, as appropriate

In total, 917 patients used statins during the follow-up, and their characteristics are listed in Table 2. We observed a significant difference in the risk factors for cardiovascular disease between statin users and non-users, and statin users also exhibited higher baseline glucose levels, a longer duration of HIV infection and a longer duration of ART. The median calendar year of statin initiation was 2011 (2009–2012) and the median duration of statin therapy was 37.3 months (IQR: 20.3–59.2 months), which accounted for 25% of the total follow-up among the statin users. The most commonly prescribed statins were rosuvastatin (*n* = 766, 83.5%) and atorvastatin (*n* = 95, 10.4%).

**Table 2:** Patients characteristics according to statins use

Characteristics	Statin users (*n* = 917)	Non-Statin users (*n* = 5278)	*p*-value
Age (years), median (IQR)	38.6 (32.3–45.9)	35.1 (30.2–41.1)	<0.001^a^
Males, *n (%)*	722 (79%)	4106 (78%)	0.55^b^
White Race, *n (%)*	881 (96%)	4909 (93%)	0.09 ^b^
Smoke, *n (%)*			<0.001 ^b^
Current/ex-smoker	395 (43%)	2088 (39%)	
Never	271 (30%)	1870 (38%)	
Unknown	251 (27%)	1320 (23%)	
Body Mass Index, *n (%)*			<0.001 ^b^
Normal (<25 kg/m2)	409 (45%)	2137 (41%)	
Overweight (25–29.9 kg/m2)	323 (35%)	1171 (22%)	
Obese (≥30 kg/m2)	100 (11%)	289 (5%)	
Unknown	85 (9%)	1681 (32%)	
HIV risk factor, *n (%)*			<0.001 ^b^
IVDU	73 (8%)	1107 (21%)	
MSM	246 (27%)	1028 (20%)	
Heterosexual	352 (38%)	1595 (30%)	
Other/Unknown	246 (27%)	1548 (29%)	
Ab-anti HCV, *n (%)*			<0.001 ^b^
Positive	136 (15%)	1564 (30%)	
Negative	721 (77%)	3143 (60%)	
Unknown	60 (7%)	570 (11%)	
HBsAg, *n (%)*			0.001 ^b^
Positive	42 (5%)	302 (6%)	
Negative	787 (86%)	4263 (81%)	
Unknown	88 (10%)	713 (14%)	
Years since first HIV-positive test, median (IQR)	18.0 (11.5–22.8)	14.1 (7.4–20.7)	<0.001^a^
AIDS diagnosis before ART, median (IQR)	96 (11%)	532 (10%)	0.72 ^b^
Nadir CD4 cell count before ART (cells/μL), median (IQR)	285 (177–398)	283 (160–412)	0.99^a^
Baseline Total Cholesterol (mg/dL), *median(IQR)*	183 (154–207) (available in 301 pts)	154 (131–179) (available in 2013 pts)	<0.001 ^a^
Baseline HDL-cholesterol (mg/dL), *median(IQR)*	38 (32–44) (available in 123 pts)	38 (32–45) (available in 939 pts)	0.56 ^a^
Baseline LDL-cholesterol (mg/dL), median (IQR)	124 (102–150) (available in 111 pts)	98 (79–117) (available in 878 pts)	<0.001 ^a^
Baseline Fasting Glucose (mg/dL), median (IQR)	88 (81–95) (available in 375 pts)	85 (79–92) (available in 2302 pts)	<0.001 ^a^
Baseline Triglycerides (mg/dL), median (IQR)	110 (82–170) (available in 318 pts)	102 (75–148) (available in 2049 pts)	<0.001 ^a^
Baseline CD4 cell count (cells/μL), median (IQR)	300 (186–433) (available in 564 pts)	307 (171–444) (available in 3119 pts)	0.75 ^a^
Baseline HIV-RNA (log_10_ copies/ml), *median(IQR)*	4.72 (4.18–5.15) (available in 421 pts)	4.74 (4.15–5.20) (available in 2584 pts)	0.85 ^a^
Baseline hemoglobin (mg/dl), median (IQR)	13.9 (12.8–14.8) (available in 403 pts)	14 (12.7–14.9) (available in 2541 pts)	0.72 ^a^
Baseline creatinine (mg/dl), median (IQR)	0.85 (0.75–0.95) (available in 287 pts)	0.81 (0.71–0.93) (available in 1952 pts)	0.017 ^a^

Baseline: date of ART initiation; if not indicated the characteristic is intended at the censoring date

^a^ by Wilcoxon rank sum test

^b^ by Chi-square or Fisher exact test, as appropriate

The incidence of DM according to statin use is reported in Table 3. Patients who developed DM exhibited a similar duration of statin treatment, compared to patients who did not develop DM (37.7 months [IQR: 20.3–60.1 months] vs. 32.7 months [IQR: 14.8–44.2 months], respectively; *P = 0.13*). High-dose statin therapy was used by 2/23 (8.7%) of the patients who developed DM, compared to by 21/894 (2.3%) of the patients who did not developed DM (*P = 0.11*).

**Table 3:** Incidence of type 2 diabetes according to statin use

Characteristic	Statins users (*n* = 917)	Non-Statin users (*n* = 5278)	*p*-value
Diagnoses of type 2 diabetes	23 (2.5%)	212 (4.0%)	0.025^a^
Person-years of follow-up (PYFU) median follow-up (IQR), years	12720 15.4 (8.9–18.1)	51429 8.9 (3.8–15.5)	<0.001^b^
Incidence rate of type 2 diabetes (95%CI) per 1000-PYFU	7.1 (4.2–10.1) ^c^	3.5 (3.0–4.0)	0.041^d^

^a^ by Chi-square test

^b^ by Wilcoxon rank-sum test

^c^ Incidence rate of statin users was calculated using only PYFU effectively spent on statin treatment while their untreated PYFU was added to PYFU of non-statin users

^d^ by univariate Fine-Gray regression model [HR = 1.58 (95%CI: 1.02–2.46)]

The results from the univariate analyses of DM risk by use of the Fine-Gray models are reported in Table 4.

**Table 4:** Univariate analyses on the risk for DM occurrence by Fine and Gray regression models

Characteristics	HR	95% CI	*p*-value
Age (per 5-years higher) ^a^	1.477	1.406–1.551	<0.001
Gender (male vs female)	2.037	1.400–2.963	<0.001
Smoking			0.28
Current/ex-smoker vs never	0.764	0.545–1.070	0.12
unknown vs never	0.904	0.572–1.429	0.67
BMI			<0.001
≥25 < 30 vs <25 kg/m^2^	1.401	1.001–1.959	0.049
>30 vs <25 kg/m^2^	2.706	1.768–4.143	<0.001
Unknown vs < 25 kg/m^2^	1.574	1.085–2.282	0.017
HIV risk factor			<0.001
IVDU vs heterosexual	0.785	0.515–1.197	0.26
MSM vs heterosexual	0.932	0.622–1.396	0.73
Other/unknown vs heterosexual	1.646	1.138–2.380	0.008
Ab-anti HCV			0.55
Positive vs negative	0.991	0.744–1.320	0.95
Unknown vs negative	1.350	0.775–2.351	0.29
HbsAg			0.14
Positive vs negative	1.608	0.998–2.593	0.051
Unknown vs negative	1.169	0.683–2.003	0.57
Years since first HIV positive test (per 5-years longer)	0.847	0.759–0.946	0.003
Nadir CD4+ cell count before ART (per 100-cells/μL higher)	0.889	0.803–0.984	0.023
AIDS diagnosis before ART (yes vs no)	1.273	0.841–1.929	0.25
Current CD4+ (per 100-cells/μL higher) ^a^	0.950	0.893–1.010	0.10
Current HIV-RNA (per log10 copies/ml) ^a^	1.261	1.113–1.428	<0.001
Current HIV-RNA ^a^			
≥50 vs <50 copies/ml	1.521	1.101–2.101	0.011
Current total cholesterol (per 50-mg/dL higher) ^a^	1.027	0.834–1.265	0.80
Current HDL-cholesterol (per 5-mg/dL higher) ^a^	0.906	0.822–0.999	0.047
Current LDL-cholesterol (per 10-mg/dL higher) ^a^	1.031	0.951–1.117	0.46
Current triglycerides (per 50-mg/dL higher) ^a^	1.061	1.047–1.076	<0.001
Current haemoglobin (per 5-g/dl higher) ^a^	1.746	1.020–2.988	0.042
Current fasting glucose (per 10-mg/dL higher) ^a^	1.159	1.136–1.184	<0.001
Current HOMA-IR (per 1-unit higher) ^a^	1.059	1.038–1.080	<0.001
Current FIB-4 (per 0.5-unit higher) ^a^	1.003	1.001–1.006	0.02
Current Framingham 10-year CVD risk (per 5% higher) ^a^	1.188	1.117–1.263	<0.001
Use of statins (yes vs no) ^a^	1.583	1.019–2.457	0.041
Calendar year of ART start (per 3-years increase)	1.133	1.047–1.227	0.002
Ever use of lamivudine (yes vs no)	0.374	0.254–0.550	<0.001
Ever use of abacavir (yes vs no)	0.516	0.393–0.677	<0.001
Ever use of emtricitabine (yes vs no)	0.163	0.119–0.225	<0.001
Ever use of tenofovir (yes vs no)	0.148	0.112–0.195	<0.001
Ever use of stavudine (yes vs no)	0.631	0.482–0.827	0.001
Ever use of zidovudine (yes vs no)	0.834	0.594–1.172	0.30
Ever use of didanosine (yes vs no)	0.453	0.345–0.595	<0.001
Ever use of efavirenz (yes vs no)	0.523	0.394–0.695	<0.001
Ever use of nevirapine (yes vs no)	0.440	0.325–0.597	<0.001
Ever use of ritonavir (yes vs no)	0.309	0.227–0.420	<0.001
Ever use of saquinavir (yes vs no)	0.586	0.442–0.777	<0.001
Ever use of lopinavir (yes vs no)	0.322	0.236–0.439	<0.001
Ever use of atazanavir (yes vs no)	0.275	0.201–0.376	<0.001
Ever use of darunavir (yes vs no)	0.182	0.110–0.301	<0.001

Abbreviations: HR, hazard ratio; 95%CI, 95% confidence interval of the hazard ratio; NE, not evaluated due to the limited number of patients treated with this drug

^a^Time-updated covariate

In the multivariate analysis (Model 1, Table 5), we observed that a lower risk of DM was associated with higher values of CD4+ cell count and exposure to abacavir, emtricitabine, tenofovir, efavirenz, nevirapine, atazanavir, darunavir. In contrast, a higher risk of DM was associated with the use of stavudine, older age, obesity, detectable HIV RNA levels, higher current triglyceride levels, higher current fasting glucose levels, higher current hemoglobin levels and shorter ART duration. Statin use was associated with a non-significant increase in the risk of DM (adjusted hazard ratio [AHR] 1.21, 95% CI: 0.71–2.07; *P = 0.47*). The statin-dependent risk of developing DM was not significantly affected by the additional inclusion of HDL-cholesterol levels (Model 2, AHR 1.58, 95% CI: 0.90–2.78; *P = 0.11*).

**Table 5:** Multivariate analysis on the risk for DM occurrence by Fine and Gray regression model (model 1; the model included 186 DM diagnoses)

Characteristics	AHR	95% CI	*p*-value
Age (per 5-years older)	1.36	1.26–1.46	<0.0001
Gender (male vs female)	1.08	0.66–1.76	0.771
Smoking			
Current/ex-smoker vs never	1.16	0.79–1.71	0.439
Unknown vs never	0.48	0.24–0.98	0.044
BMI			
≥25 < 30 vs <25 kg/m^2^	1.07	0.71–1.60	0.761
>30 vs <25 kg/m^2^	2.43	1.34–4.40	0.003
unknown vs < 25 kg/m^2^	1.02	0.57–1.82	0.954
HIV risk factor			
IVDU vs heterosexual	0.58	0.29–1.16	0.125
MSM vs heterosexual	0.96	0.56–1.63	0.877
Other/unknown vs heterosexual	1.18	0.70–1.99	0.536
Ab-anti HCV			
Positive vs negative	1.50	0.91–2.48	0.113
Unknown vs negative	0.71	0.31–1.61	0.413
Calendar year of ART initiation (per 3-years longer)	1.30	1.14–1.49	<0.0001
CD4+ (per 100-cells/μL higher) ^a^	0.91	0.84–0.99	0.025
HIV-RNA ^a^			
≥50 vs <50 copies/ml	2.00	1.41–2.84	0.0001
Triglycerides (per 50-mg/dl) ^a^	1.07	1.05–1.09	<0.0001
Haemoglobin (per 5-g/dl higher) ^a^	2.59	1.47–4.55	0.001
Fasting glucose (per 10-mg/dL higher) ^a^	1.17	1.15–1.20	<0.0001
Use of statins (yes vs no) ^a^	1.21	0.71–2.07	0.474
Ever use of lamivudine (yes vs no)	0.71	0.36–1.40	0.325
Ever use of abacavir (yes vs no)	0.53	0.36–0.77	0.0008
Ever use of emtricitabine (yes vs no)	0.30	0.17–0.54	<0.0001
Ever use of tenofovir (yes vs no)	0.48	0.29–0.81	0.006
Ever use of stavudine (yes vs no)	1.58	1.02–2.44	0.042
Ever use of zidovudine (yes vs no)	1.62	0.97–2.70	0.067
Ever use of didanosine (yes vs no)	0.98	0.66–1.46	0.932
Ever use of efavirenz (yes vs no)	0.66	0.45–0.97	0.034
Ever use of nevirapine (yes vs no)	0.36	0.23–0.56	<0.0001
Ever use of ritonavir (yes vs no)	0.95	0.60–1.53	0.845
Ever use of saquinavir (yes vs no)	0.91	0.60–1.38	0.643
Ever use of lopinavir (yes vs no)	0.76	0.38–1.02	0.064
Ever use of atazanavir (yes vs no)	0.44	0.29–0.65	<0.0001
Ever use of darunavir (yes vs no)	0.24	0.13–0.45	<0.0001

*Abbreviations*: *AHR* adjusted hazard ratio, 95%CI, 95% confidence interval of the hazard ratio

^a^Time-updated covariate

## Discussion

In the present study, we found that statin use was associated with a non-significant increase in the risk of DM occurrence among HIV-infected patients who were receiving ART. Furthermore, this increased risk became statistically significant when we considered the use of only high-dose statins. In this context, other studies have reported a dose-related diabetogenic effect for statins in the general population [12] which is probably associated to the degree of inhibition of HMG-CoA reductase activity [13]. However, there are no clear data regarding the association between statin use and DM occurrence among HIV-infected patients. For example, recent results from the randomized SATURN-HIV trial [16] revealed that the use of rosuvastatin (10 mg daily for 96 weeks) was associated with increased insulin resistance and poorer homeostatic model assessment of insulin resistance outcomes, but not with a clinical diagnosis of DM, among 72 HIV-infected patients. In line with our findings, the study by Calza et al. [17] evaluated a cohort of 3170 HIV-positive patients with a median follow-up of 5.2 years and concluded that statin use was not associated with the occurrence of DM [hazard ratio of 1.09 per year of statin exposure (95% confidence interval 0.7–1.49; *P = 0.067)*]. In contrast, a study of the HOPS cohort [18] reported a 14% increase in the incidence of DM for each year of statin use, although the authors did not specifically analyze the association between statin dose and DM occurrence. However, as the cardiovascular benefits of high-dose statin use outweigh the potential risk of developing DM [23], it may be advisable to regularly monitor for changes blood glucose and glycated hemoglobin levels in patients who are receiving high-dose statins and have a high risk of developing DM.

In the present study, DM occurrence was also related to classic risk factors for type 2 DM among HIV-infected patients, such as older age, obesity, higher plasma levels of triglycerides and glucose [15, 22, 24–26], and HCV co-infection [27, 28]. Moreover, we found that stavudine use was associated with DM occurrence. In this context, the deleterious effects of stavudine on glucose metabolism have already been described, [22, 29] and these effects appear to be secondary to mitochondrial toxicity and the occurrence of lipoatrophy, which are both related to DM occurrence [30, 31].

Interestingly we observed that a reduced risk of DM was associated with the use of abacavir, emtricitabine, tenofovir, efavirenz, nevirapine, atazanavir and darunavir. Previous studies have reported, albeit not consistently, that treatment using first-generation NRTIs or PIs may increase the risk of DM occurrence [22, 24–26, 29, 32], although there are very little data regarding the effect of more recently approved ART drugs on glucose metabolism. Nevertheless, the drugs that were associated with a reduced risk of DM in the present study are not clearly associated with ART-related risk factors for DM, such as severe mitochondrial toxicity, lipoatrophy, or lipodystrophy. Therefore, it is possible that these drugs had a relatively limited effect on glucose metabolism, compared to first-generation PIs and NRTIs [33–38].

The present study included several limitations that warrant consideration. First, the retrospective design limited the control over the previously collected data and doesn’t exclude that some residual confounding may still exists although we are confident that the major confounders were considered. Second, the assessment of the potential relationship between statin dose and incident DM provide limited evidence of this association given the limited number of patients and, for this reason, this analysis is to be intended as exploratory.

Third, it is possible that we have overestimated the effect of statins on DM occurrence, as we considered patients as statin users based on their treatment history alone, and did not consider any possibility of non-compliance with treatment. Finally, we did not have sufficient statistical power to examine the effect of different type of statins on incident DM.

Nevertheless, the present study also included several important strengths. First, we followed-up patients on a regular basis (glucose testing at least twice per year), which eliminates the possibility that we underestimated the incidence of DM. Second, our DM diagnoses were accurate, as each case was individually reviewed and confirmed to fulfil the American Diabetes Association criteria. Third, we included a large cohort of HIV-infected patients who had available data from a relatively long follow-up.

## Conclusions

In conclusion, a higher risk of DM was not associated with the use of statin but with previous use of stavudine, shorter ART duration, detectable HIV RNA levels, hypertriglyceridemia, hyperglycemia, higher hemoglobin levels, older age and obesity. In contrast, a lower risk of developing DM was associated with higher CD4+ cell counts and the use of emtricitabine, tenofovir, efavirenz, nevirapine, atazanavir and darunavir.

## Additional files

Additional file 1: Figure S1. Flow-chat of patients’ selection. (JPG 50 kb)
Additional file 2: Table S1. Exposure to antiretroviral drugs during follow-up according to occurrence of type 2 diabetes. (DOCX 15 kb)

## Abbreviations

AHR: Adjusted hazard ratio
ART: Antiretroviral therapy
BMI: Body mass index
CI: Confidence interval
DM: Type 2 diabetes mellitus
HBV: Hepatitis B virus
HCV: Hepatitis C virus
HMG-CoA: 3-hydroxy-3-methylglutaryl-coenzymeA
HR: Hazard ratio
IDD-HSR: Infectious diseases Department database at the San Raffaele Hospital
IQR: Interquartile range
IVDU: Intravenous drug use
MSM: Men who have sex with men
NRTI: Nucleoside reverse transcriptase inhibitor
PI: Protease inhibitor
PYFU: Person-years of follow-up
