# Supplementary figures and images for: Associations of statins and antiretroviral drugs with the onset of type 2 diabetes among HIV-1-infected patients
Source: BMC Infect Dis. 2017 Jan 7;17:43. doi: 10.1186/s12879-016-2099-5 (PMC5219726; doi:10.1186/s12879-016-2099-5)

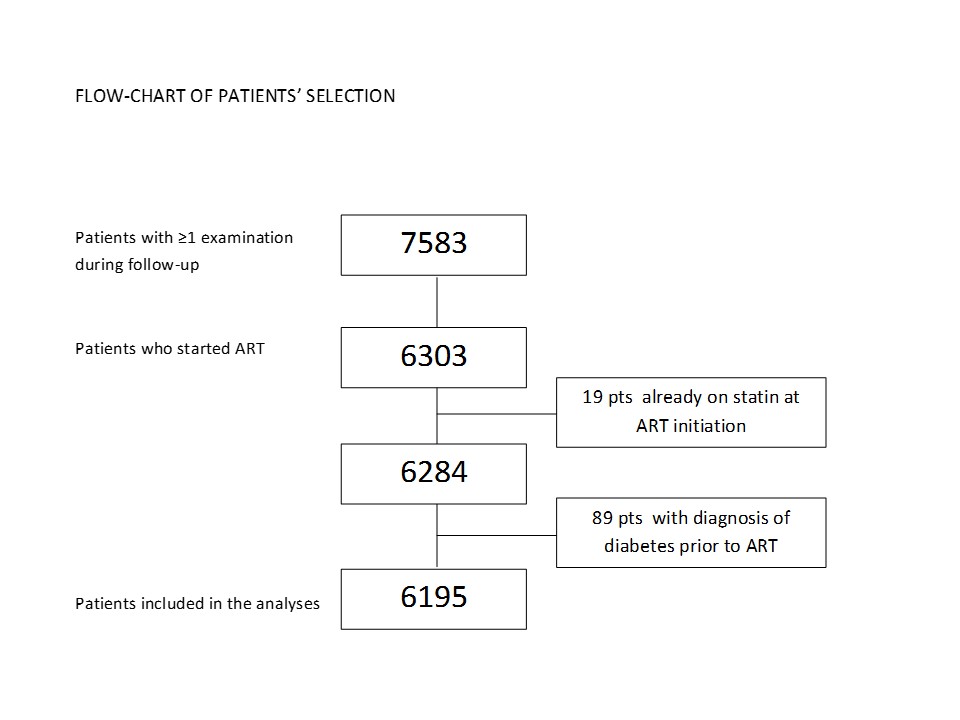

Supplement: Additional file 1: Figure S1. — Flow-chat of patients’ selection. (JPG 50 kb) [file 12879_2016_2099_MOESM1_ESM.jpg]
