# Supplementary material for: Associations of statins and antiretroviral drugs with the onset of type 2 diabetes among HIV-1-infected patients
Source: BMC Infect Dis. 2017 Jan 7;17:43. doi: 10.1186/s12879-016-2099-5 (PMC5219726; doi:10.1186/s12879-016-2099-5)
Supplement: Additional file 2: Table S1. — Exposure to antiretroviral drugs during follow-up according to occurrence of type 2 diabetes. (DOCX 15 kb) [file 12879_2016_2099_MOESM2_ESM.docx]

**Supplementary Table 1**. Exposure to antiretroviral drugs during follow-up according to occurrence of type 2 diabetes

| Characteristics | Type 2 diabetes  (n= 235) | No type 2 diabetes  (n= 5960) | p-value |
| --- | --- | --- | --- |
| Ever use of nucleoside reverse transcriptase inhibitors, *n (%)* | 227 (97%) | 5802 (97%) | 0.41^a^ |
| Ever use of lamivudine, *n (%)* | 198 (84%) | 4314 (72%) | <0.001 ^a^ |
| Ever use of abacavir, *n (%)* | 87 (37%) | 2173 (37%) | 0.89 ^a^ |
| Ever use of zalcitabine, *n (%)* | 36 (15%) | 490 (8%) | <0.001 ^a^ |
| Ever use of emtricitabine, *n (%)* | 49 (21%) | 3597 (58%) | <0.001 ^a^ |
| Ever use of tenofovir, *n (%)* | 79 (34%) | 4194 (70%) | <0.001 ^a^ |
| Ever use of stavudine (d4T), *n (%)* | 125 (53%) | 2192 (37%) | <0.001 ^a^ |
| Ever use of zidovudine (AZT), *n (%)* | 188 (80%) | 3334 (56%) | <0.001 ^a^ |
| Ever use of didanosine (DDI), *n (%)* | 99 (42%) | 2126 (36%) | 0.045 ^a^ |
|  |  |  |  |
| Ever use of non-nucleoside reverse transcriptase inhibitors, *n (%)* | 117 (50%) | 3651 (61%) | <0.001 ^a^ |
| Ever use of efavirenz, *n (%)* | 75 (32%) | 2269 (38%) | 0.06 ^a^ |
| Ever use of nevirapine, *n (%)* | 57 (24%) | 1520 (26%) | 0.70 ^a^ |
| Ever use of rilpivirine, *n (%)* | 1 (0.4%) | 616 (10%) | <0.001 ^a^ |
| Ever use of etravirine, *n (%)* | 7 (3%) | 217 (4%) | 0.72 ^a^ |
|  |  |  |  |
| Ever use of any protease inhibitor, *n (%)* | 181 (77%) | 4720 (79%) | 0.41 ^a^ |
| Ever use of ritonavir, *n (%)* | 167 (71%) | 4472 (75%) | 0.17 ^a^ |
| Ever use of tipranavir, *n (%)* | 25 (2.7%) | 80 (1.5%) | 0.012 ^a^ |
| Ever use of indinavir, *n (%)* | 83 (35%) | 1426 (24%) | <0.001 ^a^ |
| Ever use of saquinavir, *n (%)* | 78 (33%) | 1502 (25%) | 0.007 ^a^ |
| Ever use of amprenavir, *n (%)* | 17 (7%) | 525 (9%) | 0.48 ^a^ |
| Ever use of fosamprenavir, *n (%)* | 5 (2%) | 263 (4%) | 0.10 ^a^ |
| Ever use of nelfinavir, *n (%)* | 72 (31%) | 1416 (24%) | 0.019 ^a^ |
| Ever use of tipranavir, *n (%)* | 3 (1.3%) | 102 (1.7%) | 0.80 ^a^ |
| Ever use of lopinavir, *n (%)* | 57 (24%) | 2122 (36%) | <0.001 ^a^ |
| Ever use of atazanavir, *n (%)* | 53 (23%) | 2238 (38%) | <0.001 ^a^ |
| Ever use of darunavir, *n (%)* | 16 (7%) | 1384 (23%) | <0.001 ^a^ |
|  |  |  |  |
| Ever use of maraviroc, *n (%)* | 5 (2%) | 369 (6%) | 0.007 ^a^ |
| Ever use of enfuvirtide, *n (%)* | 7 (3%) | 197 (3%) | >0.99 ^a^ |
|  |  |  |  |
| Ever use of any integrase inhibitors, *n (%)* | 12 (5%) | 760 (13%) | <0.001 ^a^ |
| Ever use of raltegravir, *n (%)* | 12 (5%) | 673 (11%) | 0.002 ^a^ |
| Ever use of eviltegravir, *n (%)* | 0 | 101 (2%) | 0.034 ^a^ |
|  |  |  |  |
| Calendar year of ART start, *median (IQR)* | 1997 (1995-2000) | 2000 (1996-2007) | <0.001^b^ |
| Years since ART initiation, *median (IQR)* | 6.9 (3.1-11.8) | 10.0 (4.3-16.4) | <0.001^b^ |

a. by Chi-square or Fisher exact test, as appropriate

b. by Wilcoxon rank sum test
